# Electrolyte Effects on CO_2_ Electrochemical Reduction to CO

**DOI:** 10.1021/acs.accounts.2c00080

**Published:** 2022-06-30

**Authors:** Giulia Marcandalli, Mariana C. O. Monteiro, Akansha Goyal, Marc T. M. Koper

**Affiliations:** Leiden Institute of Chemistry, Leiden University, P.O. Box 9502, 2300 RA Leiden, The Netherlands

## Abstract

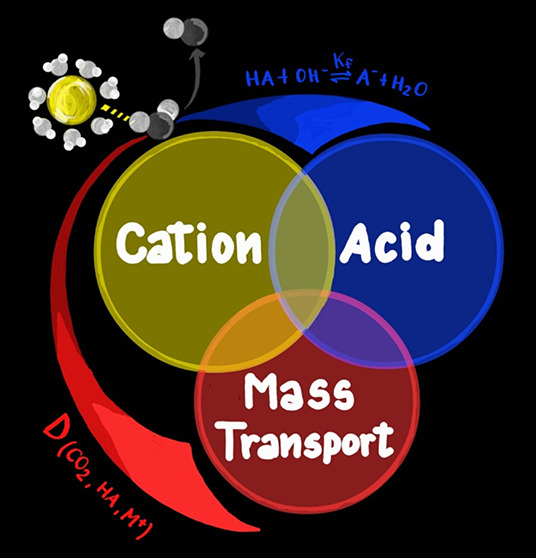

The electrochemical reduction of CO_2_ (CO2RR) constitutes an alternative to fossil fuel-based technologies for the production of fuels and commodity chemicals. Yet the application of CO2RR electrolyzers is hampered by low energy and Faradaic efficiencies. Concomitant electrochemical reactions, like hydrogen evolution (HER), lower the selectivity, while the conversion of CO_2_ into (bi)carbonate through solution acid–base reactions induces an additional concentration overpotential. During CO2RR in aqueous media, the local pH becomes more alkaline than the bulk causing an additional consumption of CO_2_ by the homogeneous reactions. The latter effect, in combination with the low solubility of CO_2_ in aqueous electrolytes (33 mM), leads to a significant depletion in CO_2_ concentration at the electrode surface.

The nature of the electrolyte, in terms of pH and cation identity, has recently emerged as an important factor to tune both the energy and Faradaic efficiency. In this Account, we summarize the recent advances in understanding electrolyte effects on CO2RR to CO in aqueous solutions, which is the first, and crucial, step to further reduced products. To compare literature findings in a meaningful way, we focus on results reported under well-defined mass transport conditions and using online analytical techniques. The discussion covers the molecular-level understanding of the effects of the proton donor, in terms of the suppression of the CO_2_ gradient vs enhancement of HER at a given mass transport rate and of the cation, which is crucial in enabling both CO2RR and HER. These mechanistic insights are then translated into possible implications for industrially relevant cell geometries and current densities.

## Key References

- GoyalA.; MarcandalliG.; MintsV. A.; KoperM. T. M.Competition between CO_2_ Reduction and Hydrogen Evolution on a Gold Electrode under Well-Defined Mass Transport Conditions. J. Am. Chem. Soc.2020, 142, (9), , 4154–41613204141010.1021/jacs.9b10061PMC7059182.^1^*Using a rotating ring disk electrode under CO_2_ reduction conditions, we determined that the water reduction branch is suppressed by increasing mass transport. It follows that improved mass transport enhances the selectivity to CO.*
- MarcandalliG.; GoyalA.; KoperM. T. M.Electrolyte Effects on the Faradaic Efficiency of CO_2_ Reduction to CO on a Gold Electrode. ACS Catal.2021, 11, (9), , 4936–49453405545410.1021/acscatal.1c00272PMC8154322.^2^*For large bulk concentrations of bicarbonate, hydrogen evolution is suppressed by sluggish mass transport. This effect is attributed to bicarbonate reduction to H_2_. In this case, the selectivity for CO increases for sluggish mass transport and low bulk cation concentration.*
- BondueC. J.; GrafM.; GoyalA.; KoperM. T. M.Suppression of Hydrogen Evolution in Acidic Electrolytes by Electrochemical CO_2_ Reduction. J. Am. Chem. Soc.2021, 143, (1), , 279–2853335620510.1021/jacs.0c10397PMC7809687.^3^*In mildly acidic media, protons at the surface are consumed by hydroxide ions generated by the interfacial CO_2_ reduction, resulting in the suppression of proton reduction reaction. Consequently, near 100% Faradaic selectivity for the electrochemical CO_2_ reduction reaction can be achieved by matching the mass transport rate of protons to the hydroxide generation rate.*
- MonteiroM. C. O.; DattilaF.; LópezN.; KoperM. T. M.The Role of Cation Acidity on the Competition between CO_2_ Reduction and Hydrogen Evolution on Gold Electrodes. J. Am. Chem. Soc.2022, 144, (4), , 1589–16023496279110.1021/jacs.1c10171PMC8815072.^4^*Through cyclic voltammetry experiments, density functional theory and ab initio molecular dynamics simulations, we find that three key parameters for CO_2_ reduction performance are ruled by cation acidity: cation accumulation at the outer Helmholtz plane, water dissociation kinetics, and cation-CO_2_ coordination.*

## Introduction

The manufacture of chemicals and fuels from waste products and renewable energy is key for the transition toward a carbon-neutral economy. In this scenario, CO_2_ is one of the main components of exhaust gases, as a result of combustion processes, and its efficient conversion back into valuable products using electricity is highly desirable. In the 1980s, Hori and co-workers brought forward the synthetic potential of the electrochemical reduction of CO_2_ (CO2RR), yielding carbon monoxide, formic acid, as well as multicarbon hydrocarbons and alcohols.^5,6^ Nowadays, among the broad range of C_1_–C_3_ products, few have reached a stage in which their generation by low-temperature CO2RR electrolysis is economically viable at the present electricity price,^7^ compared to their petrochemical counterparts. In this group falls carbon monoxide (CO), due to the optimized catalyst efficiency and high number of electrons transferred per molecular weight. Still, the low-temperature electrolysis of CO2RR to CO in aqueous electrolytes is hampered by the low energy efficiency, making high-temperature solid oxide electrolyzers more economically attractive.^8^ Besides its economical value, CO is widely recognized as the common intermediate for further reduced products.^9,10^ Starting from CO, multicarbon chemicals can be synthesized either electrochemically in consecutive reduction steps in tandem cells, or thermo-catalytically by mixing CO with H_2_ in the Fischer–Tropsch process. In CO2RR, the first electron transfer (ET) to CO_2_ (reaction 2) is generally suggested to be the rate-determining step (RDS), regardless of the final product.^10^ Given this central role of CO2RR to CO reaction step, this Account focuses on this first reductive step.

The reduction of CO_2_ to CO involves the transfer of two electrons:
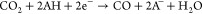
1where AH is a general acid and A^–^ its conjugated base. This electrode reaction takes place at the interface between the electrode surface and the electrolyte, where an electric double layer exists. The energetic efficiency of reaction 1 is strongly dependent on the composition of the double layer, both in terms of the nature of the electrode and the electrolyte. Gold, silver and zinc are the elemental metallic catalysts exhibiting the highest activities for CO2RR to CO.^5,11,12^ Traditionally, most research efforts addressed optimization of the catalyst to improve the system performance. Many recent studies, however, have highlighted the importance of electrolyte engineering in boosting the energy efficiency, even at practical current densities.^13−15^ For the accurate understanding of electrolyte effects, it is crucial to know the reaction mechanism. Even if there are still some controversies,^2,16,17^ the formation of CO through CO2RR is commonly proposed to happen through the following reaction intermediates:^10,18−21^
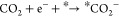
2
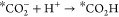
3
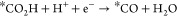
4

**Figure 1:**
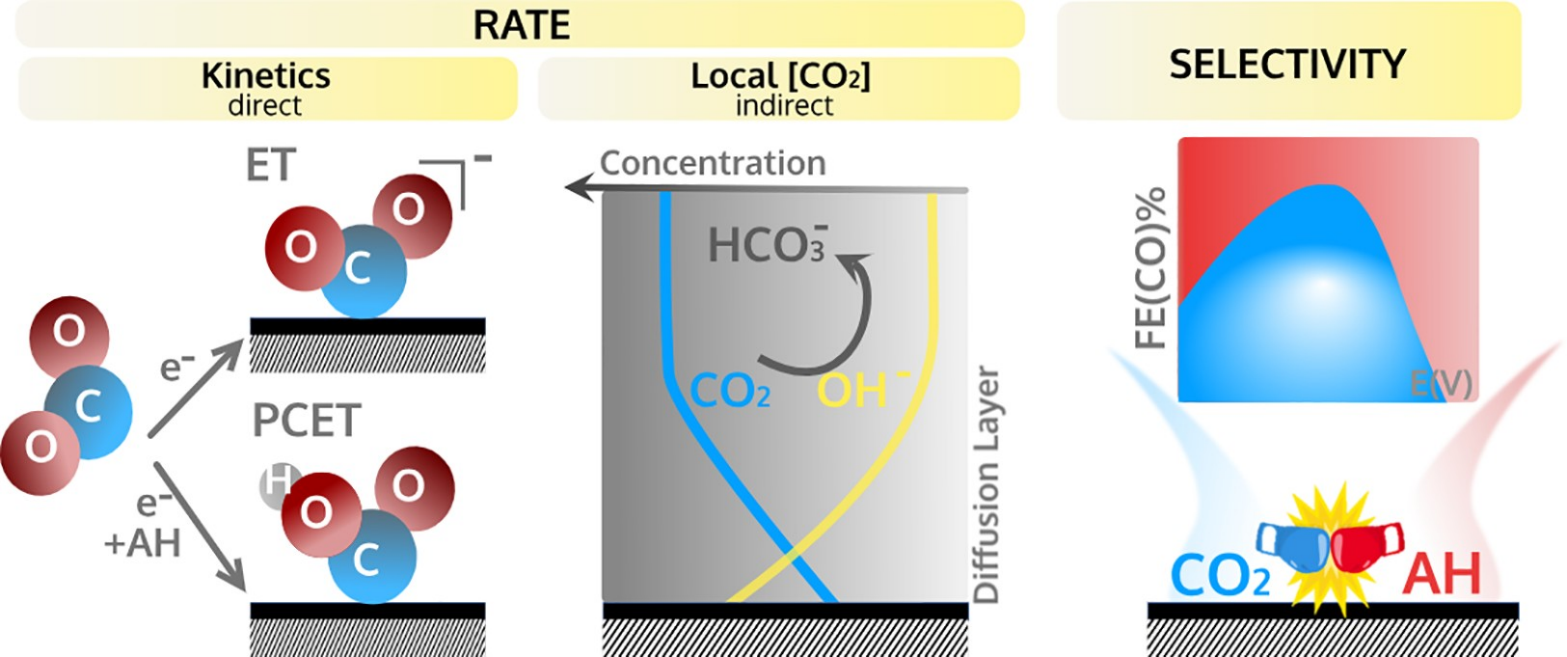
Schematic overview of the parameters (reaction rate and selectivity) considered in the discussion of electrolyte effects (AH and M^+^).

This Account aims to provide a brief but comprehensive overview of recent advancements in the understanding of the electrolyte effects on CO2RR to CO in aqueous solutions. We will outline how the nature and concentration of the electrolyte acid (AH) and cation (M^+^) affect the reaction rate and the selectivity, as illustrated in Figure 1. These effects are elucidated not only in terms of their direct influence on the CO2RR kinetics but also in terms of the indirect influence on the CO_2_ and OH^–^ concentration gradients. Therefore, we will focus on experiments performed under well-defined mass transport conditions employing online product-detection techniques.

## Setting a Framework

At the outset, it is useful to draw a framework to review literature results by examining the different ways of measuring and reporting the activity of CO2RR.

First, the choice of the analytical methodology for the quantitative product detection must be considered. Depending on the sampling time, we divide the techniques into two classes: offline techniques characterized by a large interval time between product formation and detection and online techniques characterized by fast response time (in the order of few seconds or less). Offline techniques, such as gas chromatography (GC), high-performance liquid chromatography (HPLC), and nuclear magnetic resonance (NMR), are most commonly employed. These analytical methods can be time-consuming and tend to be more affected by contaminations in the electrolytes. Having a higher time resolution and a better detection limit, online techniques are more appropriate to study the reaction mechanism as well as to screen electrode materials and electrolyte conditions. Among quantitative online techniques, we highlight techniques such as differential electrochemical mass spectrometry (DEMS)^3,22^ and rotating ring-disk electrode (RRDE) voltammetry,^23^ which we recently applied to selectively detect the amount of CO developed using a gold ring.^1,2^ It is worth mentioning the existence of other semiquantitative *in situ* techniques, spanning from simple voltammetric detection^21,24^ to more sophisticated scanning electrochemical microscopy (SECM)^21^ and infrared spectroscopy^25,26^ methods.

Second, the configuration of the electrochemical cell is a crucial parameter, as mass transport plays an important role in CO2RR. A major difference in cell design is between batch cells featuring a stagnant solution and flow cells featuring the continuous pumping of electrolyte at a given flow rate.^27^ Still, in a batch cell, mass transport may be enhanced by introducing forced convection through solution agitation (using a magnetic stirrer) or more accurately by directly controlling the rotation rate of the electrode.^1,2,23^ Significant progress has been made adopting gas diffusion electrodes (GDE) in flow cells,^28^ where a gas–solid–liquid interface is created circumventing the limitation in the decrease of interfacial CO_2_ concentration.

Concerning the preparation of electrolyte, attention should be paid to the purity grade of the chemicals, especially in fundamental studies, as metal impurities (e.g., Zn^2+^, Fe^2+^, and Pb^2+^) have been shown to mainly promote HER at the expense of CO2RR efficiency.^29,30^

Pre- and postexperimental protocols are necessary to report data in a consistent manner, simplifying comparison between literature results. In this regard, normalization to the real surface, i.e., the electrochemically active surface area (ECSA), and the choice of the potential scale (pH dependent or independent) are vital.

## Proton Donor Effects

In general, a proton donor can be any acid (AH), strong or weak, present in the solution.^2,20,32^ Hence, we refer to AH as a source of proton leading to HER according to
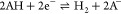
5Even if preliminary results suggest that the tendency of AH to serve as a proton donor depends on the kinetics and thermodynamics of the acid–base reaction, on the steric hindrance, and on the electrostatic effects,^2,20,32^ in neutral and alkaline pH a comprehensive description remains unclear. For simplicity, we limit the discussion to the three most relevant AH found in aqueous CO_2_-saturated solutions: hydronium (H_3_O^+^), bicarbonate (HCO_3_^–^), and water (H_2_O).

**Figure 2:**
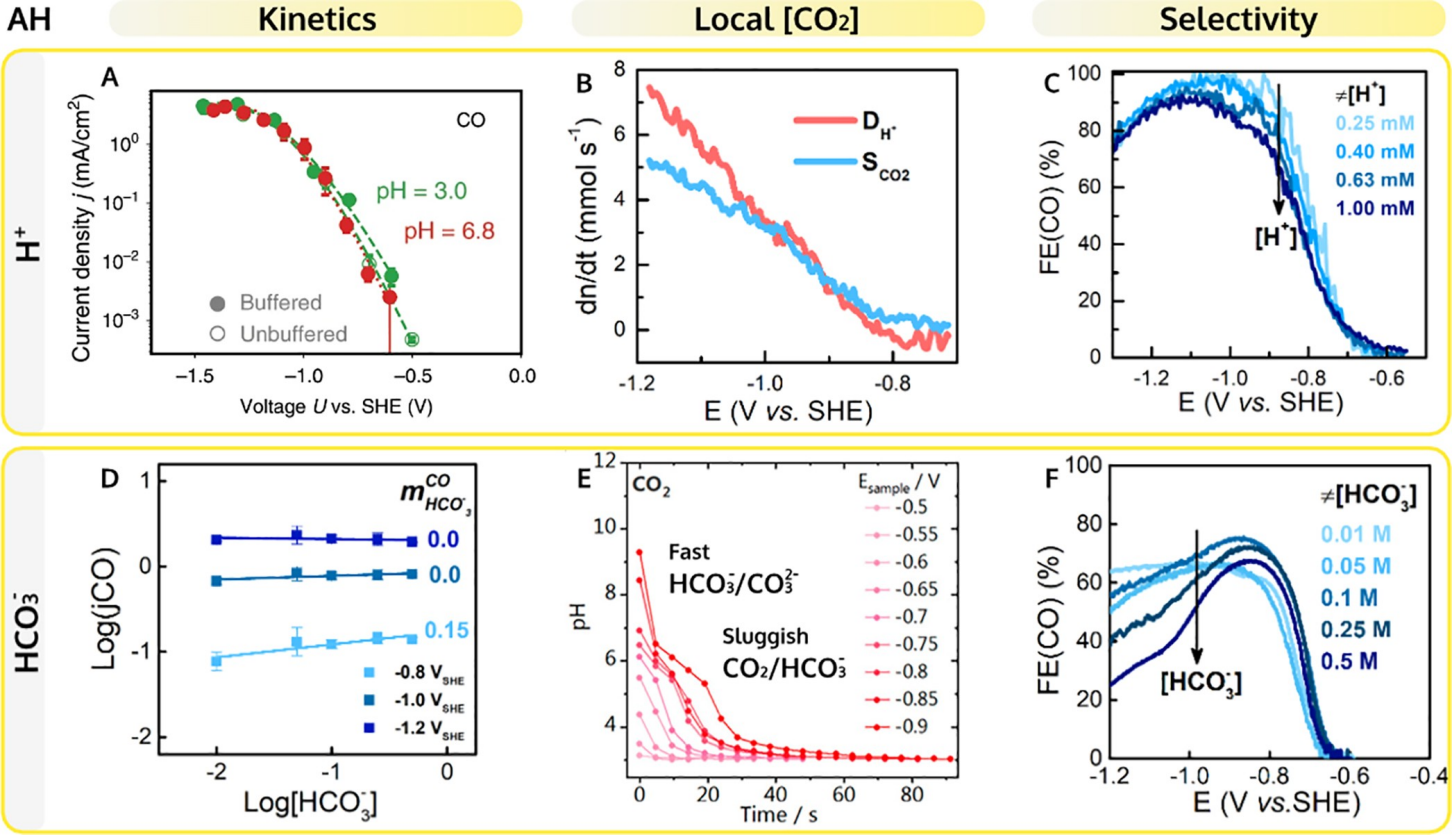
Proton donor effects. The rate of CO2RR to CO as a function of (A) proton concentration (in 0.1 M K^+^ measured by GC) and (D) bicarbonate concentration (in 0.5 M Na^+^ measured by RRDE at 2500 rpm) on Au. (B) The surplus of CO_2_ (*S*_CO_2__) and deficiency of H^+^ (*D*_H+_) due to the effect of acid–base reactions in 0.5 M NaClO_4_ purged with 0.5 bar CO_2_ at pH = 3 measured by DEMS on Au with a roughness factor of 20.3. (E) Time-dependence of the pH recovery after the reaction is “turned off” in CO_2_-saturated 0.1 M Li_2_SO_4_ pH = 3, as determined by SECM. The FE(CO) dependence on the concentration of (C) proton and (F) bicarbonate (under the same experimental conditions as in parts B and D, respectively). Panel A is adapted with permission from ref (17). Copyright 2020, Ringe et al. Springer Nature and panels B, C, D, and F from refs (2, 3, and 31). Copyright 2021 American Chemical Society.

In this section, we analyze the CO2RR rate in terms of the Tafel slope (TS) and the reaction order in the concentration of AH. These effects are evaluated on a pH-dependent potential SHE scale, where a thermodynamic shift of 59 mV per pH unit is expected for a Nernstian behavior. For a solution purged continuously with 1 atm of CO_2_, we can consider a constant bulk concentration of CO_2_ equal to 33 mM independently of the electrolyte pH and bicarbonate concentration. Generally, the experimental current due to CO2RR to CO exhibits little pH dependence^17^ (Figure 2A) and a zero reaction order in AH for both bicarbonate^2,19^ (Figure 2D) and water.^33^ This independence of the CO2RR rate on the concentration of AH together with a TS value of 120 mV dec^–1^ was interpreted as the first ET (reaction 2) being the RDS.^19,33^ However, at potentials less negative than ∼−0.8 V vs SHE, the experimental TS is ∼50 mV dec^–1^,^2,16^ suggesting that a chemical RDS is preceded by an electrochemical equilibrium. Yet, to firmly advocate a proton-coupled electron transfer (PCET) as the RDS close to the onset potential,^16,34^ a pH dependent trend of CO2RR rates should be provided. Multiscale modeling by Ringe et al. elucidated this duality in the mechanism interpretation in terms of the variations in the adsorption energy of reaction intermediates as a function of the applied potential.^17^ This was further supported by Zhu et al.^35^ The potential shift of the RDS from PCET to ET may also clarify why at a less negative potential (−0.78 Vvs SHE) a reaction order in bicarbonate of ∼1 was measured on oxide-derived Au nanoparticles.^36^ The difficulty in measuring the intrinsic kinetics of CO2RR resides not only in its potential dependence but also in the convolution with mass transport limitations,^2,17,35^ hindering the identification of AH in the electroreduction of CO_2_.

Under reductive conditions, protons are consumed and/or hydroxide ions are formed due to CO2RR (1) and HER (5), leading to an increase in the interfacial pH, in correspondence to the local current density. Since the most abundant proton donor in aqueous solution is H_2_O, we consider that the prevailing change in interfacial pH is due to the generation of OH^–^. Any AH present in solution will impact the local pH increase by reacting with the generated OH^–^ according to a simple acid–base scheme:
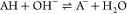
6The development of a pH gradient is very relevant as CO_2_ itself is a weak acid. CO_2_ is the main species of the bicarbonate buffer at pH lower than 6.3, but for increasing pH CO_2_ is converted into bicarbonate and then to carbonate, resulting in an additional depletion of CO_2_ at the electrochemical interface. Under stagnant conditions on a flat monocrystalline surface, Au(110), we measured a maximum CO2RR current of ∼2 mA cm^–2^ compared to a theoretical diffusion-limited current of ∼11 mA cm^–2^ at a scan rate of 10 mV s^–1^.^24^ This CO_2_ concentration gradient can be minimized by tuning the electrolyte composition (AH) and/or the mass transport, as will be discussed in the next paragraph.

To minimize the depletion of CO_2_ at the surface, reactions 8 and 9 should be favored over reaction 7.
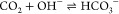
7
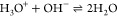
8
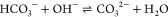
9To identify the primary solution reaction acting in the suppression of the surface alkalinity, two factors should be considered: the forward kinetic rate of the reaction between AH and OH^–^ and the diffusion rate of AH (Table 1). We recently reported that for AH = H_3_O^+^ (acidic solutions), due to the fast recombination of H^+^ and OH^–^, the proton rather than CO_2_ intercepts the locally generated OH^–^.^3^ We exclude that H^+^ is directly consumed as the proton donor in CO2RR (1) in agreement with the absence of a direct proton concentration dependence.^17^ DEMS measurements showed that OH^–^ that do not react with protons, lead to a surplus in CO_2_ consumption (Figure 2B). Similarly, the higher rate of the buffer operated by HCO_3_^–^/CO_3_^2–^ than that of CO_2_/ HCO_3_^–^ leads to a higher consumption of HCO_3_^–^ than of CO_2_. Using a SECM tip covered with a pH-sensitive molecule, one can probe the sluggish kinetics of the CO_2_/ HCO_3_^–^ buffer compared to the fast kinetics of HCO_3_^–^/CO_3_^2–^ buffer^31^ (Figure 2E). Due to the different reaction kinetics, modeling shows that the HCO_3_^–^/CO_3_^2–^ buffering is at equilibrium, while the CO_2_/HCO_3_^–^ buffer is not.^39^ Earlier work from Gupta et al. illustrated that increasing the buffer (HCO_3_^–^) capacity inhibits the increase in the interfacial pH and thereby the decrease in the local CO_2_ concentration.^38^ This simulated trend between the interfacial [CO_2_] and the bulk [HCO_3_^–^] was confirmed experimentally by Dunwell et al. using ATR-SEIRAS measurements.^25^

**Table 1:** Acidity Constants, Forward Kinetic Rate Constants of Acid–Base Reactions (Reactions 7, 8, and 9), and Diffusion Coefficients for Different AH^37,38^

AH	p*K*_a_	*k*_f_ (M s)^−1^	*D*_AH_ (m^2^ s^–1^)
CO_2_	6.3	2.23 × 10^3^	1.91 × 10^–9^
H_3_O^+^	0.0	1.4 × 10^11^	9.3 × 10^–9^
HCO_3_^–^	10.3	6.0 × 10^9^	0.92 × 10^–9^

Finally, the nature and concentration of AH also influence the FE(CO) by its effect on the rate of the competing HER. Thus, even if AH can limit the local deficiency of CO_2_, excess concentrations of AH will lead to high HER rates and low FE(CO) in the case of both proton^3^ (Figure 2C) and bicarbonate^2^ (Figure 2F). Since different branches of HER have different thermodynamics, the FE(CO) should be analyzed in terms of competition with the main HER pathway at the applied potential. Thus, for increasing AH, the decrease in the FE(CO) is ascribed to proton reduction at *E* > −1.0 V vs SHE (Figure 2C) and to bicarbonate-mediated reduction at *E* < −0.9 V vs SHE (Figure 2F). Therefore, we have recently addressed the understanding of the various parameters (pH, cation, mass transport) governing the different branches of HER, i.e., proton,^4,40^ bicarbonate,^41^ and water reduction.^40,42,43^ Significantly, in neutral/alkaline solutions water-mediated HER is strongly dependent on the cation concentration, and bicarbonate-mediated HER makes a significant contribution for bulk [HCO_3_^–^] > 0.1 M.^2,40−43^

## Cation Effects

Here, we will focus on the current understanding on the effect of metal cations on CO2RR to CO and on HER, since the latter effect often dominates FE(CO).

**Figure 3:**
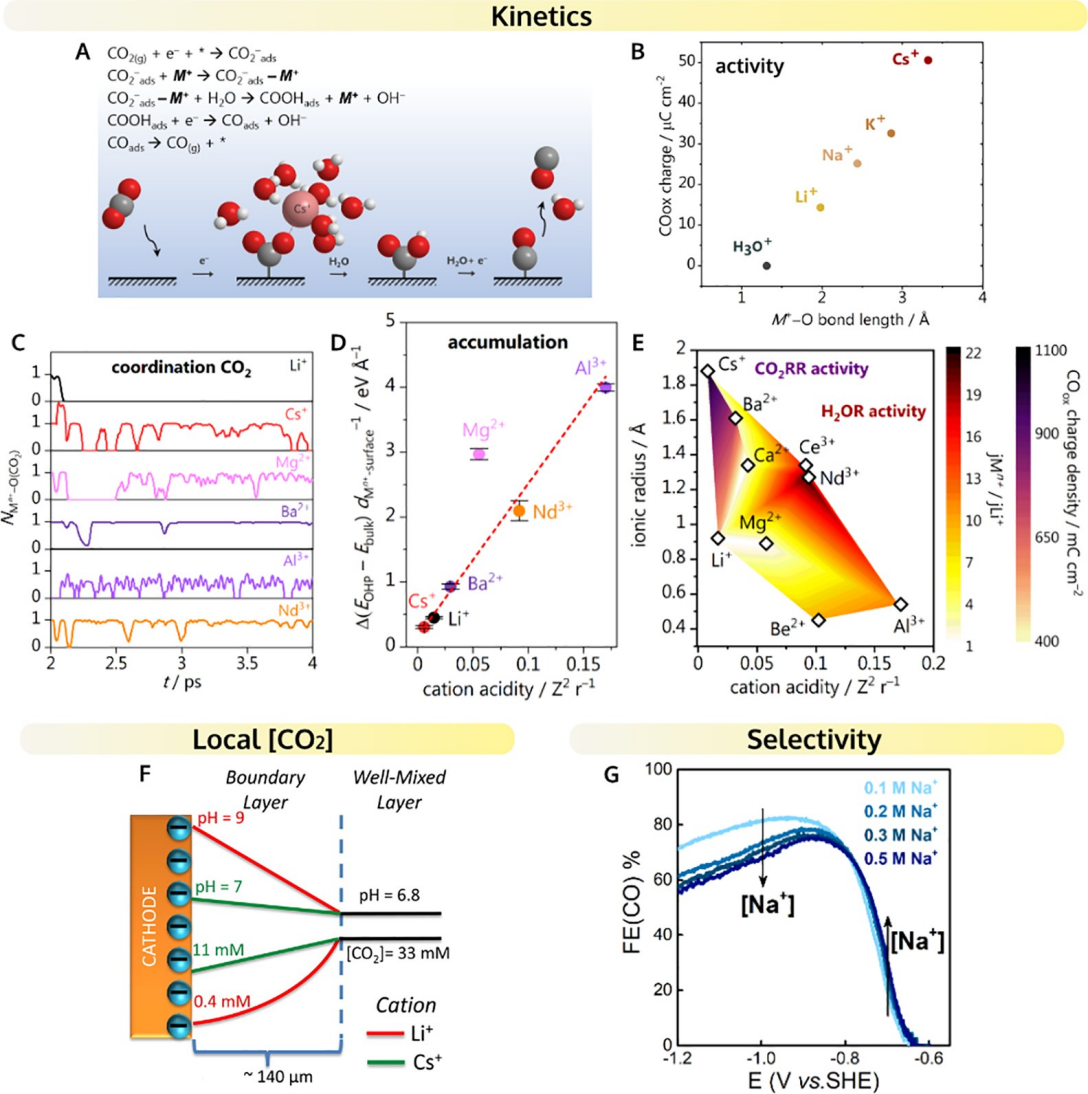
Cation effects on CO_2_ reduction to CO and HER. (A) Schematic representation of the reaction mechanism reproduced with permission from ref (21) Copyright 2021 Springer Nature. (B) CO produced on polycrystalline gold plotted as a function of the M^+^–O bond length, upon CO_2_ reduction at −1.2 V vs RHE in 1 mM M_2_SO_4_ pH = 3 with M = H, Li, Na, K, or Cs from ref (21). (C) Cation–CO_2_ coordination () for 2 ps of AIMD simulation; (D) correlation between the calculated thermodynamic driving force for cation accumulation (with respect to cation-surface distance) and cation acidity; and (E) colormap summarizing CO_2_ reduction (purple shades) and H_2_O reduction (red shades) performances at high overpotential vs cation ionic radius and cation acidity reproduced with permission from ref (4). Copyright 2021 American Chemical Society. (F) Effect of cation hydrolysis on the local pH and CO_2_ concentration reproduced with permission from ref (44). Copyright 2016 American Chemical Society. (G) FE(CO) in CO_2_-saturated 0.1 M NaHCO_3_ with different additions of NaClO_4_ as measured by gold RRDE at 2500 rpm reproduced with permission from ref (2). Copyright 2021 American Chemical Society.

Metal cations in the electrolyte are crucial to enable the CO_2_ reduction process by forming a complex with CO_2_, which favors the formation of the CO_2_^–^ intermediate.^21^ This is shown in the proposed reaction mechanism in Figure 3A. This promotion effect is so crucial that we have found that in the absence of a metal cation (in pure H_2_SO_4_ electrolyte), no CO can be formed on gold, copper, and silver electrodes. DFT-based ab initio molecular dynamics (AIMD) simulations on a Au(111)/cation/solvent system further show that partially desolvated metal cations stabilize the CO_2_ adsorption, via an explicit short-range M^+^–O(CO_2_) electrostatic interaction, which lowers the Gibbs free energy of adsorption of CO_2_ by around 0.5 eV, in comparison to solvation by water molecules only. Furthermore, metal cations decrease the O–C–O activation angle that goes from linear 180° (in water) to below 140° in the presence of a neighboring cation. In relation to this, calculated Bader charges show that coordinating cations enhance the electron transfer from the gold surface to CO_2_, from −0.50 |e^–^| in water to −0.73 |e^–^| in the presence of a neighboring Cs^+^ ion, also in agreement with the work of Huang et al.^45^ Besides this explicit short-rage interaction, the works from Chen et al.,^46^ Hussain et al.,^47^ Ringe et al.,^48^ and Liu at al.^49^ show that under CO2RR reaction conditions, cations interact with the surface via noncovalent interactions giving rise to high electric fields in the vicinity of the ion. These electrostatic potential gradients from the electrode surface toward the OHP are steeper in electrolytes containing weakly hydrated cations compared to strongly hydrated species, favoring CO2RR. Hussain et al.^47^ have shown that smaller cations increase the polarization and the polarizability of adsorbed CO (CO_ad_) and the accumulation of electronic density on the oxygen atom of CO_ad_, affecting its adsorption energy, the degree of hydrogen bonding of interfacial water, and the degree of polarization of water molecules in the cation’s solvation shell, which can influence the subsequent steps of the CO2RR. In addition, as discussed in the works of Chen et al.^46^ and Resasco et al.,^50^ cations also have a medium-range interaction with the electric dipole of the adsorbed *CO_2_^–^. This electric field effect is modulated by the electrolyte, solvation, and neighboring cations. Still, given that there is no CO2RR without cations, the interfacial electric field alone is not able to stabilize the CO_2_ adsorption and enable the reduction reaction.^21^

Beyond enabling the reaction, the metal cation identity is known to affect the reaction rate.^4,21,48,51−54^ In mildly acidic to alkaline media, or high overpotentials, more CO is produced in electrolytes containing weakly hydrated nonacidic cations (Figure 3B), while in strong acidic media weakly hydrated trivalent cations lead to the highest activity.^4,55^ The higher rates found in electrolytes containing weakly hydrated cations are, in part, due to the soft solvation shell of ions as Cs^+^, Ba^2+^, Nd^3+^, which allow these species to coordinate better with the CO_2_ molecule (Figure 3A).^4,21^ Another crucial factor that determines the effect of cations on the reaction rate is the cation concentration at the interface. The driving force for cations to accumulate at the OHP correlates linearly with cation acidity (*Z*^2^/*r*, square root of the charge over the ionic radius), thus monovalent weakly hydrated cations lead to higher near-surface concentrations (Figure 3D).^4^ The same observation has been made (for alkali cations only) in the work of Malkani et al.,^56^ Resasco et al.,^50^ and more recently Ringe et al.,^48^ in which a continuum electrolyte model is used to show that weakly hydrated cations are more concentrated at the OHP and thus induce a higher mean electronic surface charge density, favoring CO formation.

Although the rate of CO2RR to CO exhibits a clear trend with cation identity, the selectivity FE(CO) does not.^50,51^ This is due to the critical effect of cations on HER. While proton reduction is essentially cation-independent,^15,40^ on Au and Ag electrodes water reduction is promoted by alkali metal cations with a larger hydrated size, with its activity increasing from Li^+^ to Cs^+^.^43,57^ The trend between cation nature and HER activity is determined by the interfacial cation concentration,^42^ which in turn depends on the electrode identity^40^ and on the mass transport.^43^ Thus, the cation effect on FE(CO) should be carefully analyzed in view of the simultaneous effect on water reduction under given experimental conditions (electrode material and mass transport conditions). In a recent study of the role of cations (Li^+^, Cs^+^, Be^2+^, Mg^2+^, Ca^2+^, Ba^2+^, Al^3+^, Nd^3+^) on the competition between CO2RR and proton (in acidic media) and water (in neutral, alkaline media) reduction,^4^ acidic cations with a moderate hydration radius (Nd^3+^, Ce^3+^) were shown to favor CO2RR in acidic media/low overpotentials, as previously observed by Kyriacou et al.^58^ These differences come from the extreme promotional effects that acidic cations have on the water reduction reaction, only allowing these species to favor the selectivity toward CO2RR before the onset of this reaction (Figure 3E).

The cation concentration is a key parameter in determining the FE(CO). Increasing the concentration of cations promotes both CO2RR and HER, however to different extents.^2^ Systematic studies under well-defined mass transport conditions identified two distinct promotion regimes, using Na^+^ cations in bicarbonate electrolytes as a model system (Figure 3G). At low overpotentials (first regime), larger cation concentrations increase the FE(CO), in agreement with the observations of Liu at al.^49^ At more negative potentials (second regime), a high concentration of cations is more beneficial to HER, lowering FE(CO). Additional studies are necessary to understand to what extent the effect of the cation concentration on FE(CO) depends on the experimental conditions (e.g., pH, electrode roughness factor). Further work is also needed to more clearly separate the specific local effect of cations^21^ from the more global “double layer” effect attributed to cations.^48^

Besides the effects discussed in the previous paragraphs, the work of Singh et al.^44^ has shown that cations also affect the CO2RR by buffering the interfacial pH (Figure 3F). This happens due to the strong electrostatic field at the reaction interface, which decreases their p*K*_a_ of hydrolysis. Ayemoba and Cuesta^59^ and Zhang et al.^60^ confirmed this through local pH measurements using ATR-SEIRAS and RRDE, respectively. Larger cations are better buffers and during CO2RR in bicarbonate electrolyte the interfacial pH follows the trend: Li^+^ > Na^+^ > K^+^ > Cs^+^.

## Mass Transport Effects

**Figure 4:**
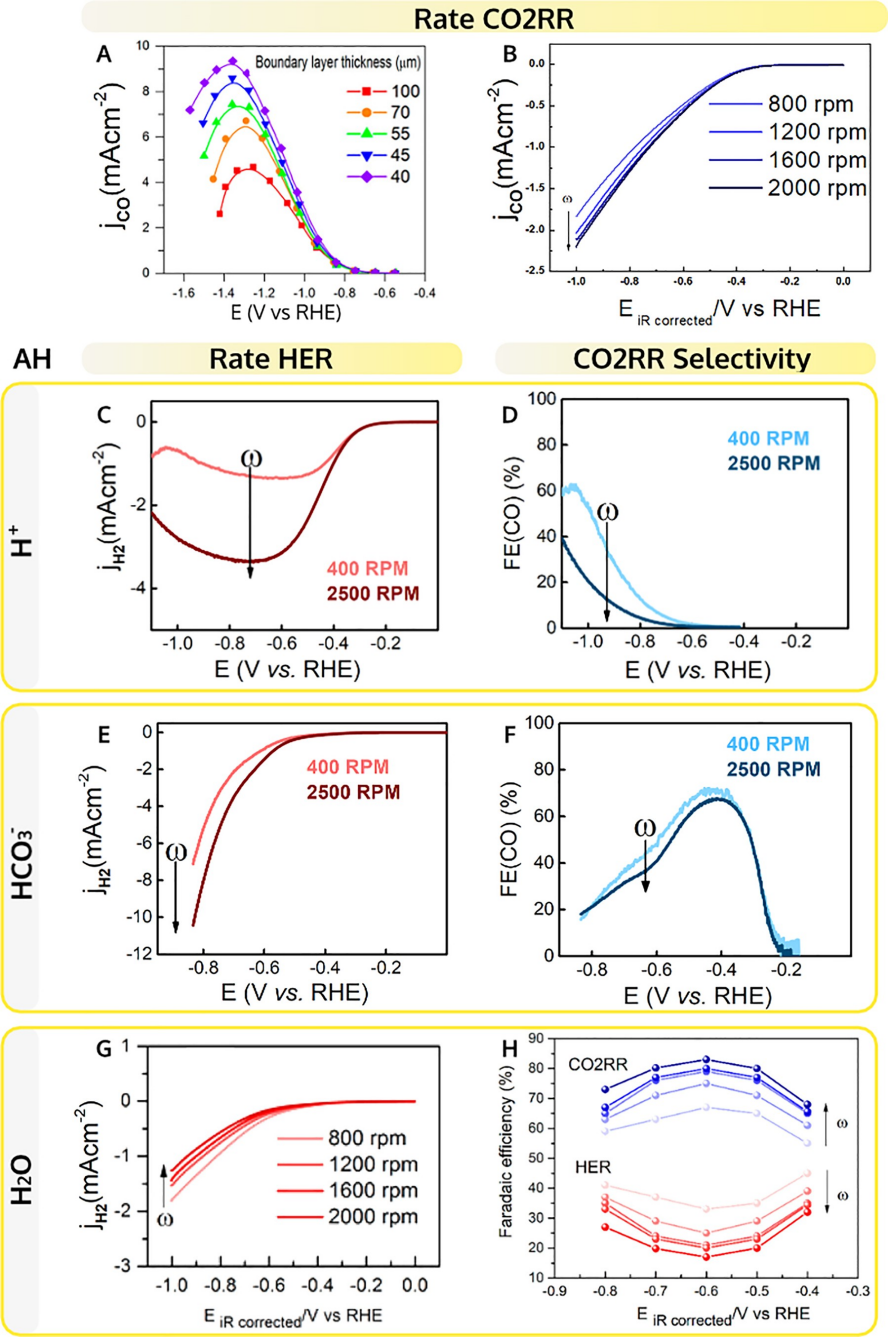
Mass transport effects. Dependence of the CO2RR to CO current on the hydrodynamic boundary layer thickness (A) determined by the CO_2_ flow rate (on Ag electrode in CO_2_-saturated 0.1 M KHCO_3_) from ref (54), Copyright 2018 American Chemical Society and (B) determined by the rotation rate (on Au RRDE in CO_2_-saturated 0.1 M NaHCO_3_) from ref (1) adapted with permission, Copyright 2020 American Chemical Society. Dependence of the HER current and the Faradaic efficiency to CO on the rotation rate on Au RRDE in CO_2_-saturated solution in the presence of different proton donors: (C,D) in 0.03 M NaClO_4_ pH 2.7 (unpublished), (E,F) in 0.5 M NaHCO_3_ from ref (2), Copyright 2021 American Chemical Society, and (G,H) in 0.1 M NaHCO_3_ from ref (1) adapted with permission, Copyright 2020 American Chemical Society.

Mass transport conditions have a profound impact on the selectivity/activity of CO2RR to CO, as they tune the concentration of different reactive and electrolyte species near the electrode–electrolyte interface. Gupta and co-workers formulated the corresponding partial differential equations and showed that under different stirring conditions, different local concentration gradients are obtained at the electrode surface.^38^ More recently, Clark and co-workers studied these effects by using a flow cell coupled to a GC^54^ as well as with a DEMS setup.^22^ Performing experiments in 0.1 M KHCO_3_, they showed that as the CO_2_ flow rate is increased, and consequently, the hydrodynamic boundary layer thickness is decreased (100 to 40 μm) and the CO formation rate is enhanced (Figure 4A). We observed a similar enhancement with a RRDE setup in 0.1 M NaHCO_3_, i.e., increasing rotation rate led to the enhancement of the CO2RR current (Figure 4B).^1^ Due to the different current densities, the enhancement observed in the studies performed by Clark and co-workers is more drastic compared to the studies done by our group. Similarly, with increasing CO_2_ partial pressure in the system, higher partial current densities for CO formation can be obtained.^3^ Measurements by ATR-SEIRAS further confirmed that a stirring rate of ≥450 rpm is enough to maintain the CO_2_ concentration at the surface close to its bulk value.^25^ Overall, the CO2RR activity is always favored by improved mass transport independently of the electrode geometry or electrolyte conditions. However, comparisons between different catalysts performances should be carried out at similar mass transport conditions.

Next, we will discuss the role of mass transport conditions in controlling the HER reaction during CO2RR and hence the overall selectivity toward CO. The role of mass transport conditions in tuning the HER rate is dependent on the nature of the proton donor itself. For example, under mildly acidic conditions, where at low overpotential the main competing reaction is proton reduction, increasing mass transport leads to the enhancement of HER at the expense of CO2RR (Figure 4C). DEMS measurements, as well as RRDE measurements (Figure 4D), show that for pH ∼ 3, slow mass transport conditions can lead to high CO Faradaic efficiencies due to the suppression of the competing HER reaction from protons.^3^ If the rate of the mass transport of the protons to the electrode is sluggish enough, the incoming protons can be homogeneously neutralized by the locally generated OH^–^ from CO2RR. Consequently, near 100% FE(CO) can be obtained in acidic media due to the complete suppression of the proton reduction reaction. This happens especially when the proton concentration is low or the proton mass transfer rate is low and/or when CO2RR rate is high, for instance, when the gold electrode has a high roughness or when the solution contains the right cation to promote the CO2RR rate^15^ (Figure 2C).

Under near-neutral pH conditions in bicarbonate containing electrolytes, the role of mass transport conditions in tuning HER is further complicated by the fact that, depending on the applied overpotential and the electrolyte composition, two distinct branches of HER can compete with CO2RR, namely, bicarbonate-mediated and water-mediated HER. These two branches show an opposite dependence on mass transport conditions.^1,2,22^ For the bicarbonate-mediated HER, increasing mass transport enhances the rate of the reaction (Figure 4E). This is understandable since enhanced mass transport can supply more HCO_3_^–^ ions to the electrode surface, thereby compensating for their paucity at the surface, both due to their reaction to form H_2_ and due to their homogeneous consumption to form carbonate ions reaction 9.^41^ Hence, at sufficiently high buffer capacity, when the bicarbonate mediated HER dominates the competition with CO2RR, an enhanced mass transport leads to lower FE(CO) due to the detrimental enhancement of HER reaction^2^ (Figure 4F).

However, the water-mediated HER reaction shows the opposite mass transport dependence compared to HER from proton and/or bicarbonate reduction. Both Bell and co-workers as well as our group have observed independently that an enhanced mass transport, either via flow rate control or via convection control, leads to the suppression of water reduction (Figure 4G).^1,22,54^ Under the conditions where water reduction dominates the overall competition with CO2RR (more negative overpotentials and lower buffer capacities ([HCO_3_^–^] ≤ 0.1 M),^2,42^ enhanced mass transport leads to the enhancement of the FE(CO) (Figure 4H).^42^ The mass transport dependence of water reduction is interesting since this reaction is expected to be independent of both mass transport and pH. However, as we have recently shown, changes in the local pH at the interface lead to corresponding changes in the local cation concentration at the interface, and cations play a central role in tuning the barrier for the rate-determining Volmer step for the water reduction reaction.^42,43^ Essentially, cations near the surface interact favorably with the dissociating water molecule at the gold surface (H_2_O + e^–^ + * + M^+^ → *H–OH^δ−^–M^+^ + (1 – δ)e^–^ → *H + OH^–^ + M^+^), thereby lowering the barrier for this RDS. In order to satisfy local electroneutrality, the increasing local pH at the surface leads to a corresponding increase in the local cation concentration, resulting in the enhancement of HER activity, which we have also shown to strongly depend on the degree of hydration of the metal cation.^40,43^

We note here that in addition to the convection conditions and electrolyte effects, introducing nanoporous structuring at the catalyst surface results in the generation of additional diffusional gradients and these gradients can be tuned by controlling pore diameter and pore length.^61−63^ In general, it has been observed that increasing porosity/roughness factor of nanoporous catalysts leads to the enhancement of FE(CO). However, the exact reason behind this enhancement is still hotly debated, as apparently conflicting activity trends have been observed for both HER and CO2RR.^64^

## Conclusions and Perspectives

**Figure 5:**
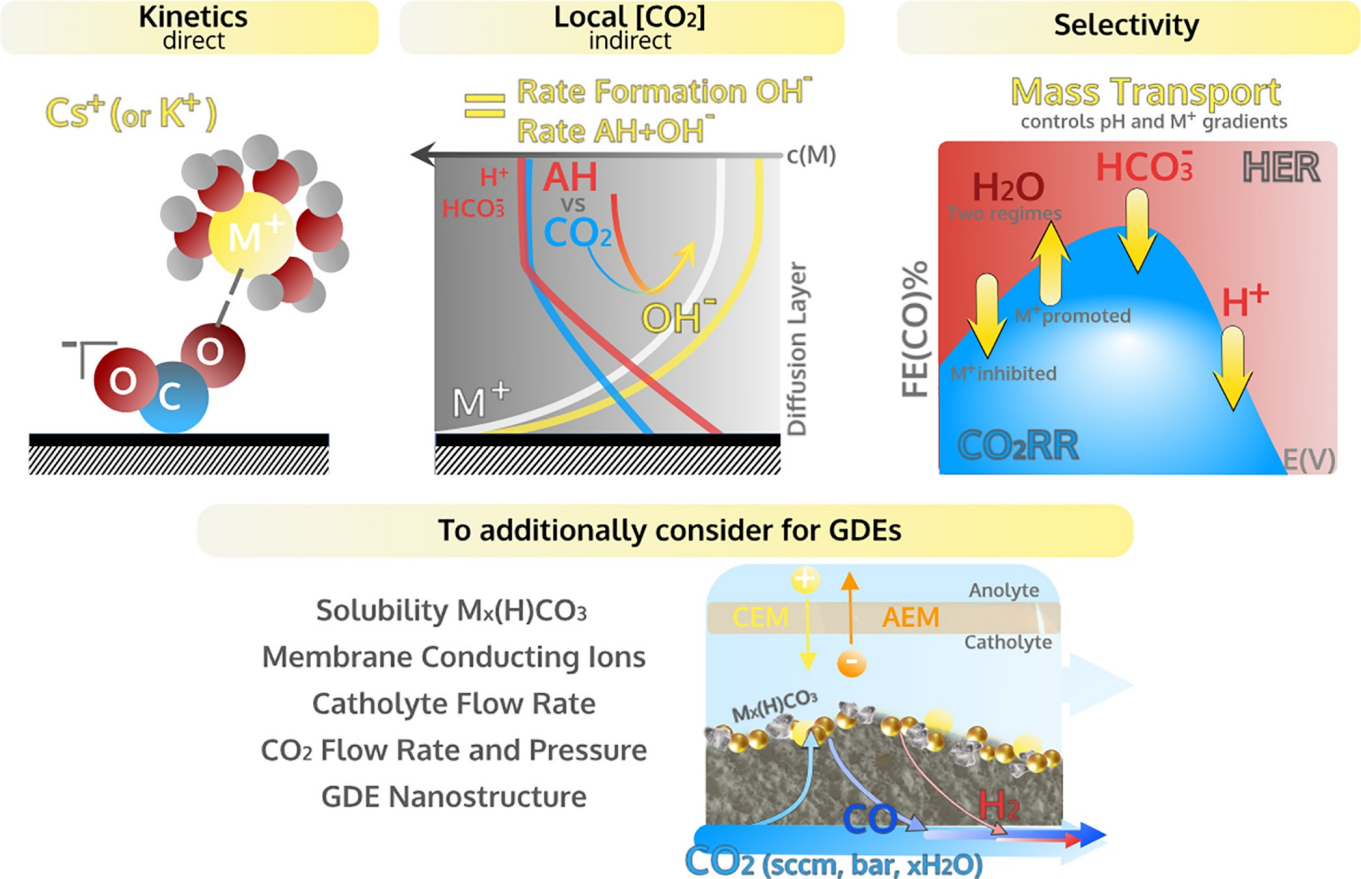
Summary of the most important electrolyte effects and design rules (in yellow) to enhance reaction rate and selectivity to CO in a batch cell. Additional principles to consider when extrapolating electrolyte effects to GDE-based CO2RR electrolyzers.

In this Account, we have described the electrolyte effects on CO2RR to CO, delineating ways to boost activity and selectivity and to rationalize and compare results obtained in a conventional three-electrode cell. In Figure 5, we summarize the reviewed concepts and contextualize these fundamental insights to electrolyte engineering for high CO2RR current density, i.e., in GDE-based electrolyzers.

Translation of the model results discussed in this brief review to practical (and more complex) geometries in real GDE electrolyzers requires much more detailed work. Nevertheless, we want to provide some (admittedly personal) perspective on potential implications, which will hopefully be useful in defining future research. CO2RR to CO requires the presence of a cation to coordinate, and hence stabilize, the first ET intermediate CO_2_^–^.^14,21^ The reaction kinetics is therefore controlled by the nature of the cation, as defined by its hydration number and acidity, among other factors.^4,21^ Weakly hydrated alkali cations like Cs^+^ (or K^+^ for a better trade-off between cost and performance) should be used to obtain high current density irrespective of the pH.^21,44,48,50^ Intermediate concentration of cations (≤0.1 M) should be preferred, because cations also promote the concomitant water and bicarbonate reduction.^2,41−43^ The partial current density to CO also benefits from the presence of multivalent acidic cations like Nd^3+^, as long as the potential is less negative than the onset of water reduction.^4^

Experiments performed with a GDE have confirmed the essential role of the cation to enable CO2RR,^14^ along with elucidating why flow electrolyzers commonly display higher current densities than electrolyzers based on membrane electrode assembly (MEA).^13,65^ Specifically, the presence of a catholyte, explicitly the cation, in the former system, and the absence in the latter one (if not accordingly tailored^13,66^). Analogously, in a GDE setup, the partial current density for CO production increases with the electrolyte cation in the order Li^+^ < Na^+^ < K^+^ < Cs^+^.^13,15,52,65^ Besides the inherent effect of cations on the CO2RR kinetics, in GDE-based electrolyzers the cation dependence is interlinked with the higher conductivity and higher solubility of (bi)carbonate salts of weakly hydrated alkali cations.^15,67^ Even if highly concentrated alkaline solutions (≥1.0 M) appear to enhance the initial CO current density due to the higher conductivity,^65,68^ in the long term they cause more salt formation at the cathode and should be avoided. Therefore, the use of a lower concentration (≤1.0 M) of Cs^+^- or K^+^-containing alkaline solutions is preferred to obtain better performance in the long run.^13,67^ Major precipitation of salts at high current density has also been shown to hamper the employment of multivalent cations for CO2RR electrolyzers.^69^ Nevertheless, Endrődi et al. have successfully demonstrated that electrolyte engineering can overcome this dual role of cations: improving the reaction kinetics but also favoring salt formation.^13^ By using a pure water anolyte and periodically flushing the catholyte of a zero-gap cell with a solution containing 1.0 M CsOH with 25 v/v% isopropanol, they could maintain the “activating” role of the cation while hindering the deposition of (bi)carbonate on the GDE and thus reach high CO current densities (∼450 mA cm^–2^) over a long time period (200 h).

The proton donor does not significantly influence CO2RR electrokinetics, but it does indirectly influence it by limiting the consumption of CO_2_ by the homogeneous reactions. Importantly, under CO2RR conditions, the HER activity with proton and bicarbonate as AH can be minimized by matching the rate of their homogeneous consumption with the formation rate (the geometrical current) of OH^–^.^2,3^ Overall, a lower fraction of CO_2_ is converted into (bi)carbonate, and a lower near-surface availability of AH drives a conspicuous enhancement of the FE(CO). Also mass transport plays a salient role in defining concentration gradients. To optimize unwanted consumption of CO_2_ by homogeneous reactions, it is important to tune the concentration of AH and the mass transport conditions to the geometric current density, i.e., to the ECSA of the cathode. To neutralize the same local alkalinity increase, a lower bulk concentration of protons compared to bicarbonates is needed due to the higher diffusion coefficient of protons. For a current density on the order of 1 mA cm^–2^ (flat electrodes), a low concentration of proton (pH 3–4) and bicarbonate (∼0.01 M) is preferred. Roughly, for an increase in CO2RR current of 1 order of magnitude, the concentration of proton and bicarbonate should increase correspondingly, e.g., for 10 mA cm^–2^ pH 2 and 0.1 M HCO_3_^–^. A key conclusion of recent work is that mass transport mainly affects HER and CO2RR much less. Therefore, in acidic and bicarbonate-containing solutions, sluggish mass transport conditions are preferred.^2,3^ When water is the main proton donor of HER, its activity can be altered by varying the cation and its interfacial concentration.^40,43^ The water reduction activity is suppressed by increasing mass transport at mild current densities and cation concentrations;^1^ however, we expect the opposite mass transport dependence for very high interfacial cation concentrations due to local crowding.^43^

Although in a GDE, CO_2_ gas is directly fed to the catalyst, bypassing a deficiency in the reactant local concentration, achieving high carbon mass balance to the desired product is a central theme to curtail operating costs.^70^ The carbon efficiency can be measured in terms of CO_2_ single-pass conversion, which is the percentage of CO_2_ converted to the desired reduced product (e.g., CO) divided by the total CO_2_ input. This value is small, around 10% or lower, for alkaline-catholyte electrolyzers with an anion exchange membrane (AEM), despite the extremely high initial energy efficiency attributed to the high conductivity of OH^–^.^65,67,68,70^ Regardless of the starting alkaline pH, the final solution in the catholyte is mainly composed by (bi)carbonate ions generated by the reaction of CO_2_ with OH^–^.^70^ Because most of the studies assess the electrolyzer performance on a short-time scale (few minutes to 1 h), they avoid the buildup of (bi)carbonate salts convoluting the extrapolation of long-term relevant electrolyte-dependence. When aiming for prolonged operational time, the ubiquitous presence of (bi)carbonate in the system should be taken into account in the choice of the membrane. Endrődi et al. reported a large improvement in the energy efficiency at 1 A cm^–2^ while preserving high CO_2_ conversion efficiency up to 45% by adopting an anion exchange membranes (AEM) exhibiting high conductivity for carbonate together with a small thickness.^71^ Notably, an even larger enhancement in the CO_2_ conversion up to ∼80% was achieved by introducing protons in the electrolyte^14^ or in a custom-designed MEA^72^ to locally regenerate the CO_2_. On the other hand, the excess protons induced an important drop in CO2RR selectivity with the FE(H_2_) being ∼40%.^14,72^ However, we recently showed that choosing the “right” cation, even in acidic electrolyte, a FE(CO) of ∼90% can be obtained using Cs^+^, while at the same current density it remains close to 0% using Li^+^.^15^ This promising outlook needs to be further validated on a prolonged operational time (10 h or longer).

Alternatively, through ingenious cell design, the solution bicarbonate can be acidified to generate *in situ* the CO_2_. Lees et al. have demonstrated the viability of liquid-fed bicarbonate electrolyzers using a cation exchange membrane (CEM) to provide locally the proton source.^73^ This configuration offers the advantage to cut upstream cost by avoiding the CO_2_ regeneration step. On the other hand, the kinetics of the acid–base reactions may also pose an upper limit on the maximum CO_2_ reduction rate. Specifically, at large current densities, we expect that the high formation rate of OH^–^ triggers the fast neutralization by H^+^ and HCO_3_^–^ limiting in this way the slower reaction between H^+^ and HCO_3_^–^ to generate CO_2_.

Even if in a GDE the hydrodynamics and the interface is different compared to a batch cell, the mass transport conditions affect to a large extent the single-pass CO_2_ conversion and the selectivity.^74^ The mass transport can be tuned by separately varying the flow rate and pressure of the CO_2_ gas feed and the flow rate of the catholyte. High catholyte flow rates are more efficient in removing the as-generated OH^–^ and crucially boosting the reaction rate of CO.^65^ Increasing the CO_2_ flow rate enhances the FE(CO) at the expense of an inferior CO_2_ single-pass conversion.^75^ The optimized CO_2_ flow rates for both energy and mass efficiency are in the range of 10–20 sccm.^65,74^

Finally, the ultimate goal of CO2RR electrolysis has been shifting from being exclusively driven by high current density, as set by water electrolysis, to conciliate energy and carbon efficiency. This prospect is based not only on operational expenses but also on the consequential decrease in (bi)carbonate salt formation, which is partially responsible for the mediocre durability of CO2RR electrolyzers.^76^ Electrolyte engineering offers a route to combine the increase in energy efficiency with durability for commercial implementation.^13^ Key is the identity and concentration of the metal cation to favor CO2RR kinetics, combined with mass transport and electrolyte (concentration of AH) design to manage the carbon balance. The flow of species should be modeled as a function of various mass transport parameters (i.e., catholyte and CO_2_ gas flow rates) and electrode geometry to find the optimum concentration of AH and M^+^ for a given current density.
